# Core Warming of Coronavirus Disease 2019 Patients Undergoing Mechanical Ventilation: A Pilot Study

**DOI:** 10.1089/ther.2023.0030

**Published:** 2023-11-30

**Authors:** Nathaniel P. Bonfanti, Nicholas M. Mohr, David C. Willms, Roger J. Bedimo, Emily Gundert, Kristina L. Goff, Erik B. Kulstad, Anne M. Drewry

**Affiliations:** ^1^ Department of Emergency Medicine, University of Texas at Southwestern Medical Center, Dallas, Texas, USA.; ^2^ Department of Emergency Medicine, University of Iowa Carver College of Medicine, Iowa City, Iowa, USA.; ^3^ Department of Critical Care, Sharp Memorial Hospital, San Diego, California, USA.; ^4^ Department of Internal Medicine, Division of Infectious Disease, VA North Texas Health Care System and University of Texas Southwestern Medical Center, Dallas, Texas, USA.; ^5^ Department of Anesthesiology, University of Texas at Southwestern Medical Center, Dallas, Texas, USA.; ^6^ Department of Anesthesiology, Washington University School of Medicine, St. Louis, Missouri, USA.

**Keywords:** COVID-19, esophageal warming, induced hyperthermia, sepsis, respiratory insufficiency, virus replication

## Abstract

Fever is a recognized protective factor in patients with sepsis, and growing data suggest beneficial effects on outcomes in sepsis with elevated temperature, with a recent pilot randomized controlled trial (RCT) showing lower mortality by warming afebrile sepsis patients in the intensive care unit (ICU). The objective of this prospective single-site RCT was to determine if core warming improves respiratory physiology of mechanically ventilated patients with coronavirus disease 2019 (COVID-19), allowing earlier weaning from ventilation, and greater overall survival. A total of 19 patients with mean age of 60.5 (±12.5) years, 37% female, mean weight 95.1 (±18.6) kg, and mean body mass index 34.5 (±5.9) kg/m^2^ with COVID-19 requiring mechanical ventilation were enrolled from September 2020 to February 2022. Patients were randomized 1:1 to standard of care or to receive core warming for 72 hours through an esophageal heat exchanger commonly utilized in critical care and surgical patients. The maximum target temperature was 39.8°C. A total of 10 patients received usual care and 9 patients received esophageal core warming. After 72 hours of warming, the ratio of arterial oxygen partial pressure to fractional inspired oxygen (PaO2/FiO2) ratios were 197 (±32) and 134 (±13.4), cycle thresholds were 30.8 (±6.4) and 31.4 (±3.2), ICU mortalities were 40% and 44%, 30-day mortalities were 30% and 22%, and mean 30-day ventilator-free days were 11.9 (±12.6) and 6.8 (±10.2) for standard of care and warmed patients, respectively (*p* = NS). This pilot study suggests that core warming of patients with COVID-19 undergoing mechanical ventilation is feasible and appears safe. Optimizing time to achieve febrile-range temperature may require a multimodal temperature management strategy to further evaluate effects on outcome. ClinicalTrials.gov Identifier: NCT04494867.

## Introduction

Fever is a recognized protective factor in patients with sepsis (Rumbus et al., 2017). Prospective data have shown that afebrile patients have higher 28-day mortality (37.5% vs. 18.2%), increased acquisition of secondary infections (35.4% vs. 15.9%), and suppressed Human Leukocyte Antigen-DR isotype expression suggestive of monocyte dysfunction over time (Drewry et al., 2018). Elevated temperatures have been shown to augment immune function, increase production of protective heat shock proteins, directly inhibit microorganism growth, reduce viral replication, and enhance antibiotic effectiveness (Drewry and Hotchkiss, 2013; Launey et al., 2011).

The first pilot randomized controlled trial (RCT) of warming as a therapy for sepsis found lower mortality by warming afebrile patients in the intensive care unit (Drewry et al., 2022). Conversely, RCTs have failed to find benefits to reducing fever of infectious etiology (Dallimore et al., 2018; Drewry et al., 2017; Gozzoli et al., 2001; Peters et al., 2019; Schulman et al., 2005; Young et al., 2019; Young et al., 2015; Zhang, 2015), and therapeutic hypothermia in sepsis has been found to be either of no benefit, or harmful (Itenov et al., 2018; Saoraya et al., 2020).

Innate and adaptive immunological processes appear to be accelerated by fever (Evans et al., 2015; Lee et al., 2012; Peters et al., 2019). More rapid recovery from chickenpox (Doran et al., 1989), malaria (Brandts et al., 1997), and rhinovirus (Stanley et al., 1975) infections have been shown by avoiding antipyretic medication. Because many viruses replicate more robustly at cooler temperatures, such as those found in the nasal cavity (33–35°C) than at warmer core body temperature (37°C) (Chan et al., 2011; Foxman et al., 2015; Laporte et al., 2019; Ping, 2003; Zou et al., 2020), elevated temperature may offer another benefit in treating viral illnesses.

The virus causing coronavirus disease 2019 (COVID-19), severe acute respiratory syndrome coronavirus 2 may behave similarly to other viruses susceptible to temperature changes (Wang et al., 2020). The fact that fever has often abated by the time a COVID-19 patient requires mechanical ventilation may offer a specific window of opportunity for treatment with therapeutic hyperthermia (Arabi et al., 2022; Chan et al., 2011; Foxman et al., 2015; Roger, 2021; Zhou et al., 2020).

The first RCT of warming therapy in sepsis found that by using forced-air warming, not all patients were able to achieve target temperature (Drewry et al., 2022). Additional means of providing heat to patients may, therefore, be desirable. The provision of heat at the patient's core (where viral replication may be greatest) rather than through peripheral heat transfer across the skin may offer further mechanistic benefits. A dedicated device (ensoETM; Attune Medical, Chicago, IL) offers a means to provide heat through the esophagus using a closed-loop flow of water that can be adjusted to cause temperature change (Furrer et al., 2022). Toward this goal, the aim of this pilot study was to investigate the feasibility of providing febrile-range temperatures to COVID-19 patients requiring mechanical ventilation through core warming, to determine if core warming improves respiratory physiology of mechanically ventilated patients with COVID-19, allowing earlier weaning from ventilation, and greater overall survival.

## Materials and Methods

This was a single-center randomized controlled pilot trial (NCT04494867), and we have previously published the study protocol (Bonfanti et al., 2020). Participants were included if they were aged ≥18 years, diagnosed with COVID-19, intubated for respiratory failure, and had a maximum baseline temperature of <38.3°C. We excluded patients with a contraindication to core warming using an esophageal core warming device, pregnancy, body weight <40 kg, a Do-Not-Resuscitate order, history of acute stroke, postcardiac arrest, or multiple sclerosis. Written informed consent was obtained from each patient's legally authorized representative before enrollment. The randomization algorithm was built into the electronic data capture system, Redcap, which was used to collect data in the study.

Once the randomization form was entered into the system and saved, the back-end algorithm ran and participants were assigned an arm corresponding to either study device (Group A—Core warming) or standard of care (Group B—Control). Patients underwent randomization after being enrolled into the study and before the placement of the core warming device. Randomization was performed in a 1:1 manner and maintained on the limited access, encrypted, Redcap database. All procedures followed were in accordance with the ethical standards of the Sharp Memorial Hospital IRB (IRB No. 2007901; approved August 17, 2020) and with the Helsinki Declaration of 1975.

The primary outcome was the severity of acute respiratory distress syndrome as measured by the ratio of arterial oxygen partial pressure to fractional inspired oxygen (PaO2/FiO2) ratio. Secondary outcomes included the change in viral load measured in lower respiratory tract samples, the duration of mechanical ventilation, and mortality. Patient core warming was provided by a commercially available esophageal heat exchange system (ensoETM; Attune Medical). The device was set to 42°C after initiation and maintained at 42°C for the duration of 72 hours of treatment unless patient temperature exceeded 39.8°C, at which point the device setpoint was reduced to 40°C. Control patients were provided standard temperature management.

## Results

A total of 19 patients were randomized (10 patients to control, 9 to core warming). The patients had a mean age of 60.5 (±12.5) years, 37% were female, with a mean weight of 95.1 (±18.6) kg, and a mean body mass index 34.5 (±5.9) kg/m^2^. Patient baseline demographics by group are as shown in Table 1.

**Table 1.** Baseline Demographics

Variable	Control (*n* = 10)	Warming (*n* = 9)	*p*
Age, years (median) Missing	59.4 (10.3) 0	61.7 (15.1) 0	0.690
Time to enrollment (days) Missing	9.1 (11.8) 0	9.4 (13.1) 0	0.953
Weight, kg Missing	95.2 (14.2) 0	94.8 (23.5) 0	0.961
Body mass index, kg/m^2^ Missing	33.4 (3.4) 0	35.8 (7.8) 0	0.380
Female Missing	3 (30) 0	4 (44) 0	0.650
Tobacco Missing	1 (11) 1	1 (14) 2	0.999
Ethanol Missing	3 (38) 2	1 (20) 4	0.999
Initial SOFA score Missing	8.5 (2.3) 4	9.5 (1.7) 5	

SOFA, Sequential Organ Failure Assessment score.

**FIG. 1.**
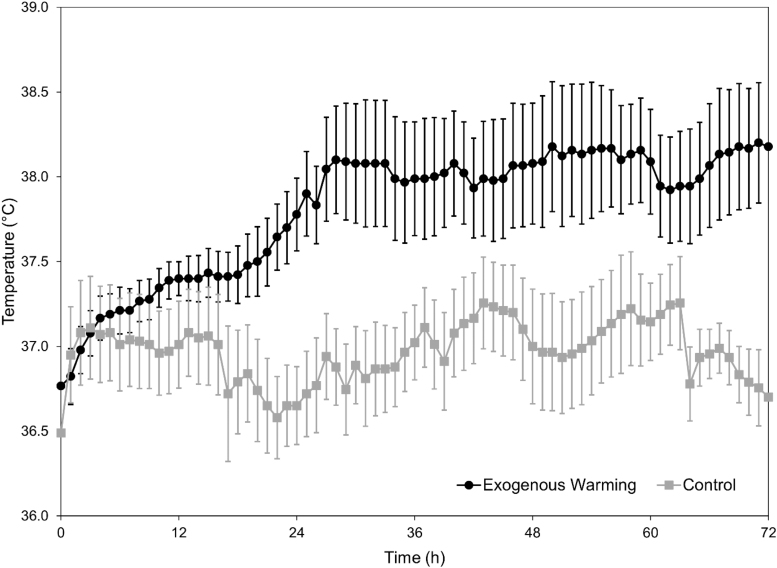
Patient temperature curves over 72 hours of treatment.

Temperature curves for each patient group are shown in Figure 1. Good separation between groups was obtained.

Measured outcomes are shown in Table 2. The outcomes were similar in this small pilot study. There was a single adverse event attributed to the use of the device, reported as an episode of nausea, vomiting, and abdominal pain.

**Table 2.** Outcomes

Variable	Control (*n* = 10)	Warming (*n* = 9)	*p*
Cycle threshold, 72 hours Missing	30.8 (6.4) 3	31.4 (3.2) 3	0.852
Delta-cycle threshold Missing	4.4 (1.4) 4	0.2 (2.0) 4	0.115
ICU mortality	4 (40) 0	4 (44) 0	0.999
30-Day mortality	3 (30) 0	2 (22) 0	0.999
PaO2/FiO2 at *t* = 0	165 (19.4)	119 (11.6)	
PaO2/FiO2 at *t* = 24 hours	155 (21.2)	130 (17.0)	
PaO2/FiO2 at *t* = 48 hours	197 (30.6)	158 (19.4)	
PaO2/FiO2 at *t* = 72 hours	197 (32.0)	134 (13.4)	

ICU, intensive care unit; PaO2/FiO2, the ratio of arterial oxygen partial pressure to fractional inspired oxygen.

## Discussion

Growing data support beneficial effects from warming patients with severe infections, and there are increasing research efforts being undertaken to better understand the mechanistic underpinnings for the clinical effects reported. Challenges in warming patients to febrile-range hyperthermia will need to be adequately addressed to fully explore hyperthermia as a treatment. This pilot study suggests that core warming provided by a commercially available esophageal heat transfer system appears safe and can provide febrile-range temperatures to patients with COVID-19 undergoing mechanical ventilation.

Core body temperature is far more tightly regulated than other physiological parameters (including blood pressure and heart rate), and because of the existence of robust thermal defense mechanisms, warming patients to above normal temperatures can be difficult (Sessler, 2009). Previous data have found that core warming with a dedicated esophageal heat transfer device is able to provide warming in patients with particular difficulty in maintaining normothermia, such as burn patients undergoing surgery (Furrer et al., 2022). At present, it remains unclear if a shorter time to a febrile target temperature is beneficial or will be required to demonstrate benefits seen in early clinical studies.

A single adverse event attributed to the use of the device was reported as an episode of nausea, vomiting, and abdominal pain. No differences in sedation level were noted between cohorts in this study, and all patients received sedation levels sufficient to maintain mechanical ventilation, making this event unlikely to be related to sedation level, but a clear etiology or definitive attribution to the device remains uncertain. COVID-19 is known to have gastrointestinal symptoms, which makes it difficult to rule out as a factor (Kwei-Nsoro et al., 2023). To date >50,000 uses have been completed for cooling or warming applications in critical care, trauma, burn surgery, and electrophysiology, without evidence of an association with nausea, vomiting, or abdominal pain (Anderson et al., 2022; Furrer et al., 2022; Leung et al., 2023).

Further studies are in development, which aim to investigate further mechanistic underpinnings behind the effects of elevated temperature in severe infections. Given the known challenges in warming patients to above-normal body temperature, a multimodal temperature management strategy may be required, and is being anticipated, in subsequent investigations.

Limitations of this study include the small sample size, the inability to blind health care providers treating enrolled patients, and the lack of measurement of specific immune factors such as interleukin (IL)-1, IL-6, tumor necrosis factor, and interferon.

## Conclusions

Core warming of patients with COVID-19 undergoing mechanical ventilation is feasible and appears safe. To optimize time to achieve febrile-range temperature in subsequent studies, a multimodal temperature management strategy may be necessary.

## References

[B1] Anderson CM, Joseph C, Fisher R, et al. Targeted temperature management using esophageal cooling. Ther Hypothermia Temp Manag 2022;12(4):235–239; doi: 10.1089/ther.2022.0033. 36301260 PMC9700367

[B2] Arabi YM, Myatra SN, Lobo SM. Surging ICU during COVID-19 pandemic: An overview. Curr Opin Crit Care 2022;28(6):638–644; doi: 10.1097/mcc.0000000000001001. 36226716 PMC9612411

[B3] Bonfanti N, Gundert E, Drewry AM, et al. Core warming of coronavirus disease 2019 (COVID-19) patients undergoing mechanical ventilation—A protocol for a randomized controlled pilot study. PLoS One 2020;15(12):e0243190; doi: 10.1371/journal.pone.0243190. 33259540 PMC7707531

[B4] Brandts CH, Ndjave M, Graninger W, et al. Effect of paracetamol on parasite clearance time in Plasmodium falciparum malaria. Lancet 1997;350(9079):704–709; doi: 10.1016/s0140-6736(97)02255-1. 9291905

[B5] Chan KH, Peiris JS, Lam SY, et al. The effects of temperature and relative humidity on the viability of the SARS coronavirus. Adv Virol 2011;2011:734690; doi: 10.1155/2011/734690. 22312351 PMC3265313

[B6] Dallimore J, Ebmeier S, Thayabaran D, et al. Effect of active temperature management on mortality in intensive care unit patients. Crit Care Resusc 2018;20(2):150–163. 29852854

[B7] Doran TF, De Angelis C, Baumgardner RA, et al. Acetaminophen: More harm than good for chickenpox? J Pediatr 1989;114(6):1045–1048; doi: 10.1016/s0022-3476(89)80461-5. 2656959

[B8] Drewry AM, Ablordeppey EA, Murray ET, et al. Antipyretic therapy in critically ill septic patients: A systematic review and meta-analysis. Crit Care Med 2017;45(5):806–813; doi: 10.1097/CCM.0000000000002285. 28221185 PMC5389594

[B9] Drewry AM, Ablordeppey EA, Murray ET, et al. Monocyte function and clinical outcomes in febrile and afebrile patients with severe sepsis. Shock 2018;50(4):381–387; doi: 10.1097/SHK.0000000000001083. 29240644 PMC5999533

[B10] Drewry AM, Hotchkiss RS. Counterpoint: Should antipyretic therapy be given routinely to febrile patients in septic shock? No. Chest 2013;144(4):1098–1101; doi: 10.1378/chest.13-0918. 24081340 PMC3787912

[B11] Drewry AM, Mohr NM, Ablordeppey EA, et al. Therapeutic hyperthermia is associated with improved survival in afebrile critically ill patients with sepsis: A pilot randomized trial. Crit Care Med 2022;50(6):924–934; doi: 10.1097/ccm.0000000000005470. 35120040 PMC9133030

[B12] Evans SS, Repasky EA, Fisher DT. Fever and the thermal regulation of immunity: The immune system feels the heat. Nat Rev Immunol 2015;15(6):335–349; doi: 10.1038/nri3843. 25976513 PMC4786079

[B13] Foxman EF, Storer JA, Fitzgerald ME, et al. Temperature-dependent innate defense against the common cold virus limits viral replication at warm temperature in mouse airway cells. Proc Natl Acad Sci USA 2015;112(3):827–832; doi: 10.1073/pnas.1411030112. 25561542 PMC4311828

[B14] Furrer F, Wendel-Garcia PD, Pfister P, et al. Perioperative targeted temperature management of severely burned patients by means of an oesophageal temperature probe. Burns 2022;49(2):401–407; doi: 10.1016/j.burns.2022.03.015. 35513952

[B15] Gozzoli V, Schottker P, Suter PM, et al. Is it worth treating fever in intensive care unit patients? Preliminary results from a randomized trial of the effect of external cooling. Arch Intern Med 2001;161(1):121–123; doi: 10.1001/archinte.161.1.121. 11146708

[B16] Itenov TS, Johansen ME, Bestle M, et al. Induced hypothermia in patients with septic shock and respiratory failure (CASS): A randomised, controlled, open-label trial. Lancet Respir Med 2018;6(3):183–192; doi: 10.1016/s2213-2600(18)30004-3. 29325753 PMC10928558

[B17] Kwei-Nsoro R, Attar B, Shaka H, et al. Independent predictors and causes of thirty-day gastrointestinal readmissions following COVID-19-related hospitalizations: Analysis of the National Readmission Database. Gastroenterol Res 2023;16(3):157–164; doi: 10.14740/gr1623. PMC10284648 37351083

[B18] Laporte M, Stevaert A, Raeymaekers V, et al. Hemagglutinin cleavability, acid stability, and temperature dependence optimize influenza B virus for replication in human airways. J Virol 2019;94(1):e01430−e01419; doi: 10.1128/jvi.01430-19. 31597759 PMC6912116

[B19] Launey Y, Nesseler N, Mallédant Y, et al. Clinical review: Fever in septic ICU patients—Friend or foe? Crit Care (London, England) 2011;15(3):222; doi: 10.1186/cc10097. PMC3218963 21672276

[B20] Lee CT, Zhong L, Mace TA, et al. Elevation in body temperature to fever range enhances and prolongs subsequent responsiveness of macrophages to endotoxin challenge. PLoS One 2012;7(1):e30077; doi: 10.1371/journal.pone.0030077. 22253887 PMC3254634

[B21] Leung LWM, Toor P, Akhtar Z, et al. Real-world results of oesophageal protection from a temperature control device during left atrial ablation. Europace 2023;25(5):euad099; doi: 10.1093/europace/euad099. 37096813 PMC10228621

[B22] Peters MJ, Woolfall K, Khan I, et al. Permissive versus restrictive temperature thresholds in critically ill children with fever and infection: A multicentre randomized clinical pilot trial. Crit Care (London, England) 2019;23(1):69; doi: 10.1186/s13054-019-2354-4. PMC6407208 30845977

[B23] Ping CK. Rapid response to: Graphic Outbreak of severe acute respiratory syndrome in Hong Kong Special Administrative Region: Case report. BMJ 2003;326:850. 12702616 10.1136/bmj.326.7394.850 PMC153470

[B24] Roger C. COVID-19: Should we consider it as a septic shock? (The treatment of COVID-19 patients in the ICU). Curr Opin Anaesthesiol 2021;34(2):119–124; doi: 10.1097/aco.0000000000000956. 33470663

[B25] Rumbus Z, Matics R, Hegyi P, et al. Fever is associated with reduced, hypothermia with increased mortality in septic patients: A meta-analysis of clinical trials. PLoS One 2017;12(1):e0170152; doi: 10.1371/journal.pone.0170152. 28081244 PMC5230786

[B26] Saoraya J, Musikatavorn K, Puttaphaisan P, et al. Intensive fever control using a therapeutic normothermia protocol in patients with febrile early septic shock: A randomized feasibility trial and exploration of the immunomodulatory effects. SAGE Open Med 2020;8:2050312120928732; doi: 10.1177/2050312120928732. 32547753 PMC7271676

[B27] Schulman CI, Namias N, Doherty J, et al. The effect of antipyretic therapy upon outcomes in critically ill patients: A randomized, prospective study. Surg Infect (Larchmt) 2005;6(4):369–375; doi: 10.1089/sur.2005.6.369. 16433601

[B28] Sessler DI. Thermoregulatory defense mechanisms. Crit Care Med 2009;37(7 Suppl.):S203–S210; doi: 10.1097/CCM.0b013e3181aa5568. 19535948

[B29] Stanley ED, Jackson GG, Panusarn C, et al. Increased virus shedding with aspirin treatment of rhinovirus infection. JAMA 1975;231(12):1248–1251. 163931

[B30] Wang W, Xu Y, Gao R, et al. Detection of SARS-CoV-2 in different types of clinical specimens. JAMA 2020;323(18):1843–1844; doi: 10.1001/jama.2020.3786. 32159775 PMC7066521

[B31] Young P, Saxena M, Bellomo R, et al. Acetaminophen for fever in critically ill patients with suspected infection. N Engl J Med 2015;373(23):2215–2224; doi: 10.1056/NEJMoa1508375. 26436473

[B32] Young PJ, Bellomo R, Bernard GR, et al. Fever control in critically ill adults. An individual patient data meta-analysis of randomised controlled trials. Intensive Care Med 2019;45(4):468–476; doi: 10.1007/s00134-019-05553-w. 30741326

[B33] Zhang Z. Antipyretic therapy in critically ill patients with established sepsis: A trial sequential analysis. PLoS One 2015;10(2):e0117279; doi: 10.1371/journal.pone.0117279. 25710375 PMC4339198

[B34] Zhou F, Yu T, Du R, et al. Clinical course and risk factors for mortality of adult inpatients with COVID-19 in Wuhan, China: A retrospective cohort study. Lancet 2020;395(10229):1054–1062; doi: 10.1016/S0140-6736(20)30566-3. 32171076 PMC7270627

[B35] Zou L, Ruan F, Huang M, et al. SARS-CoV-2 viral load in upper respiratory specimens of infected patients. N Engl J Med 2020;382(12):1177–1179; doi: 10.1056/NEJMc2001737. 32074444 PMC7121626

